# Resilience and stress as predictors of work engagement: the mediating role of self-efficacy in nurses

**DOI:** 10.3389/fpsyt.2023.1202048

**Published:** 2023-08-15

**Authors:** Elard Cabrera-Aguilar, Margarita Zevallos-Francia, Mardel Morales-García, Andrés Alexis Ramírez-Coronel, Sandra B. Morales-García, Liset Z. Sairitupa-Sanchez, Wilter C. Morales-García

**Affiliations:** ^1^Unidad de Posgrado en Salud Pública, Escuela de Posgrado, Universidad Peruana Unión, Lima, Peru; ^2^Unidad de Posgrado en Salud, Escuela de Posgrado, Universidad Peruana Unión, Lima, Peru; ^3^Nursing Career, Azogues Campus, Catholic University of Cuenca, Cañar, Ecuador; ^4^Laboratory of Psychometry, Comparative Psychology and Ethology, Catholic University of Cuenca, Cuenca, Ecuador; ^5^Health and Behavior Research Group (HBR), Catholic University of Cuenca, Cuenca, Ecuador; ^6^Departamento Académico de Enfermería, Obstetricia y Farmacia, Facultad de Farmacia y Bioquímica, Universidad Científica del Sur, Lima, Peru; ^7^Escuela Profesional de Psicología, Facultad de Ciencias de la Salud, Universidad Peruana Unión, Lima, Peru; ^8^Escuela de Medicina Humana, Facultad de Ciencias de la Salud, Universidad Peruana Unión, Lima, Peru; ^9^Escuela de Posgrado, Universidad Peruana Unión, Lima, Peru; ^10^Facultad de Teología, Universidad Peruana Unión, Lima, Peru

**Keywords:** resilience, stress, work engagement, self-efficacy, nurses

## Abstract

**Background:**

Nurses face high levels of stress and work demands, which can affect their work engagement and psychological well-being. Resilience and self-efficacy have been identified as important resources to improve nurses’ adaptation and work engagement.

**Objective:**

This study aimed to evaluate the mediating role of self-efficacy in the relationship between resilience and stress on work engagement in Peruvian nurses.

**Methods:**

A cross-sectional design was used, and data were collected from a sample of 459 nurses. Self-report questionnaires were administered to measure self-efficacy, resilience, stress, and work engagement. SEM analyses were performed to examine the relationship between these variables, and a mediation analysis was conducted to evaluate the role of self-efficacy as a mediator in the relationship between resilience, stress, and work engagement.

**Results:**

The results indicated a positive relationship between resilience, self-efficacy, and work engagement, as well as a negative relationship between stress and work engagement. Additionally, self-efficacy mediated the relationship between resilience and work engagement, as well as the relationship between stress and work engagement in nurses.

**Conclusion:**

Personal resources such as self-efficacy are a key factor in the relationship between resilience (work resources), stress (work demands), and work engagement of Peruvian nurses. Strengthening self-efficacy and resilience can improve work engagement and personal satisfaction of nurses. Hospital administrators and nursing managers should consider the importance of resilience, stress, work engagement, and self-efficacy in registered nurses and develop effective strategies to improve them. This can have a positive impact on the quality of care provided to patients and on the job satisfaction of nurses.

## 1. Introduction

Nursing staff shortage is a significant problem that can affect healthcare quality and lead to poor patient care outcomes, ineffective teamwork, and decreased work performance. To address this issue and improve the quality of healthcare services, it is necessary to promote professional commitment, nursing competence maintenance, and self-efficacy enhancement (1, 2). Healthcare organizations seek committed and dedicated employees who can successfully face challenges (3). Work engagement is crucial to achieve effective and efficient healthcare delivery, and nurses, as the backbone of the healthcare system, play an important role in ensuring this (4). However, the stressful nature of nursing work puts nurses at risk of emotional distress, including burnout, depression, anxiety, secondary traumatic stress, and suicide. Nurses are exposed to a wide variety of stressors, including trauma, shift work, workplace violence, and resource insufficiency (5, 6). In the context of mental health, nurses are exposed to unique stressors, such as seeing patients self-harm and caring for patients who may attempt or complete suicide (7). In this context, resilience and stress are important predictors of nurses’ work engagement (8). Self-efficacy plays a significant mediating role in this relationship, and future research should examine other factors that may influence nurses’ engagement, such as social support and personality. To improve nurses’ work engagement, it is necessary to address the challenges they face and promote their resilience and self-efficacy.

The job demands-resources model (JD-R) is an important framework for understanding the relationship between work-related well-being and stress, as well as engagement and performance in nurses (9). This model considers that work resources are the best predictors of individual and organizational engagement and performance through a motivational process (10). It also highlights the role of workers’ job resources, such as the positive evaluation or belief of control workers have over their environment, as it is positively related to engagement and performance and also reduces the negative impact of work demands like stress (11). Adequate work resources can effectively balance the various task requirements during work so that individuals can maintain a good work state, leading to high work engagement (9).

In terms of work resources, the literature has emphasized the importance of workers’ self-efficacy, that is, their beliefs in their ability to control their own functioning (9). When nurses believe in their abilities to perform clinical tasks skillfully, they tend to perceive work requirements as challenges to overcome rather than threats to avoid (12). Previous studies have shown that high levels of self-efficacy are associated with higher levels of work engagement in nurses (13, 14). Self-efficacy determines the amount of work and effort invested in tasks, and if it is high, nurses dedicate more time and energy to a task, become more involved, and concentrate more easily (15). Therefore, research focuses on finding ways to alleviate the negative impact of interpersonal stressors in the workplace (16–18). The JD-R model suggests the importance of work resources in combating an exhausting work environment (19). Work resources refer to the psychological capacities that enable individuals to be flexible and adaptable to exhausting resource circumstances (20). Previous research suggests that the extent to which work demands result in emotional exhaustion depends on the amount of personal resources (21). Self-efficacy is an important factor that influences the perception of work demands and, therefore, work engagement in nurses. The higher the self-efficacy, the less they will be affected by stress, and the more likely they are to engage in their work. In addition, resilience also plays an important role in work engagement. Resilience refers to an individual’s ability to recover from stressful situations and maintain good work performance. The higher the resilience, the easier it is for nurses to adapt to challenges and maintain high work engagement (22, 23).

Thus, the JD-R model provides a useful framework for understanding the relationship between self-efficacy, resilience, stress, and work engagement in nurses. The literature suggests that work resources, such as self-efficacy and resilience, are important in mitigating the negative impact of stress and improving work engagement. Therefore, the objective of this research is to evaluate the mediating role of self-efficacy in the relationship between resilience, stress, and work engagement.

### 1.1. Literature review

#### 1.1.1. Work engagement

Work engagement is a crucial aspect for workers’ well-being and organizational success. It is a positive and motivating work-related state characterized by vigor, dedication, and absorption (24). It has been extensively studied and shown to be related to positive aspects such as health (25), happiness (26), satisfaction (27), and favorable behaviors for the organization, such as personal initiative (28), active learning (29), and customer satisfaction (30). Work engagement is reflected in a range of positive outcomes for both workers and organizations. It increases job satisfaction (15), decreases psychological strain, and improves performance (31). In the case of nurses, a high level of work engagement has been associated with a decrease in turnover intentions, delays, and absenteeism (32), as well as an improvement in emotional health (33, 34). In addition, work engagement has a positive impact on work efficiency, quality of care, and patient satisfaction, which in turn reflects organizational outcomes (35).

Within the Job Demands-Resources model (JD-R), it has been shown that job resources are positively related to work engagement (36). Self-efficacy, an important personal resource, is also positively related to work engagement (4). Factors influencing nursing competence include effective self-management and professional commitment. Low work engagement results in various organizational outcomes such as high turnover rates, low job satisfaction, and low performance (37, 38).

#### 1.1.2. Stress

Stress is a significant problem in the nursing profession, and it has been identified as a factor contributing to job dissatisfaction and staff turnover (39). Stress is a complex psychobiological process that is experienced when an individual perceives a threat or danger in their environment (40). Nurses face a variety of stressful situations, including the stresses of patients and the demands of their families (41), which can affect their professional performance and lead to burnout (42). Despite the challenges, the nursing profession can also be a source of satisfaction and well-being for workers (43). The JD-R model is a useful framework for assessing the antecedents of work-related stress. Job demands include physical, psychological, social, or organizational aspects that require physical and psychological effort or skills. The JD-R is a balance model, as the perception of the adequacy of job resources acts as a buffer against the negative impacts of job demands perceived as high. This model is relevant for stress management in high-demand environments, such as nursing work (36, 44). The perceptions and attitudes of nurses about their work are crucial because they have a high turnover rate, which can disrupt continuity of care and increase costs. Numerous studies have focused on work-related stress and burnout among nursing staff because they work in high-stress environments. This has detrimental effects on their mental and physical health, productivity, and job effectiveness and can lead to absenteeism (2, 45).

#### 1.1.3. Resilience

Resilience is the ability of an individual to recover from or successfully confront adverse situations (46). Resilience has been described as both a personality trait (47) a dynamic process (48). Resilience is defined as an individual’s ability to recover quickly and easily from setbacks that occur in their life (49). Strength is a common theme in various definitions of resilience, and people who are described as resilient are able to persist and overcome challenging obstacles (50). Nurses may be negatively impacted in their resilience due to the emotional labor of suppressing emotions during interactions with patients (51). Moral distress, which occurs when a person is unable to act in accordance with their core values due to internal and external constraints, may contribute to professional burnout (7, 52). The nature of nurses’ role, which involves providing continuous care and forming close relationships with patients and families, puts them at greater risk of compassion fatigue and professional burnout (53). However, protective factors have been identified that enable nurses to positively adapt in stressful work situations, such as personal resilience (54, 55). Maintaining psychological well-being and mental health are common indicators of the resilient process after adverse events (56). Most people are exposed to regular stressors and one or more life-threatening experiences throughout their lifetime (57). Understanding what facilitates resilience and positive adaptation can play an important role in improving people’s mental health in many contexts. Resilience can be viewed as both a personality trait, a process, and an outcome (58). When considered as a personality trait, resilience is fixed and stable over time, while, when viewed as a dynamic process, resilience can develop throughout life and vary according to context and time (59). Resilience in nurses has been studied from the perspective of the JD-R model. Studies have found that personal resilience can act as a work resource to cope with job demands and reduce the risk of professional burnout and compassion fatigue (60–62). Additionally, resilience can also help nurses maintain their psychological well-being and mental health in adverse situations, which is a common indicator of the resilient process (56).

#### 1.1.4. Self-efficacy

Workplace self-efficacy is a key component of personal resources, and refers to an individual’s beliefs about their competence and ability to perform their job (63). Positive self-efficacy is associated with self-directed motivation, energy, and positive expectations of success, based on the belief in one’s competence and ability (64). Employees with strong workplace self-efficacy likely have the motivational and psychological skills to withstand difficult work situations, which would otherwise deplete their emotional resources and energy. These employees may perceive incivility in the workplace as less threatening and feel less emotionally exhausted. The cognitive-social theory defines self-efficacy as the belief in one’s own abilities to achieve specific successes in the future (17). Research has shown that positive self-efficacy is a predictor of positive states, such as work engagement, especially in demanding work environments (65). Bandura’s social cognitive theory (SCT) (63) asserts that self-efficacy beliefs influence behavior, thought, and feeling. For example, people tend to choose tasks they believe they can do and avoid those they consider too difficult. People with low self-efficacy tend to exaggerate their deficits and produce negative thoughts that lead to stress and hinder their ability to utilize available resources (66). Empirical research has shown that positive self-efficacy is a predictor of positive states such as work engagement through positive spirals, especially in demanding work environments (67, 68).

The Job Demands-Resources (JD-R) model recognizes job demands as aspects of work that require sustained physical and/or psychological effort and are associated with physiological and/or psychological costs (69). This model emphasizes the role of personal resources of workers, such as self-efficacy, in job engagement and performance, as they are positively related and can reduce the negative impact of job demands (9). Self-efficacy is considered a personal resource that can help individuals cope with job demands and enhance their motivation and commitment, as it refers to an individual’s belief in their capabilities to control their environment and perform a task or achieve a specific goal (70). Self-efficacy can act as a mediator between job demands and job engagement, buffering the negative impact of demands and enhancing the positive effects of job challenges (14, 71, 72). Initially, job demands were thought to deplete energy and be linked to burnout and exhaustion. However, subsequent studies have identified that job demands can be both negative stressors and positive challenges (73, 74). Nurses with high levels of self-efficacy perceive their work environment as a place where they are capable of effectively facing challenges and feeling more prepared to cope with job demands (39).

Furthermore, professional self-efficacy can help professionals address demanding challenges, creating higher demands on practicing nurses to demonstrate their caregiving skills (75). In this sense, challenging job demands can have a positive impact on self-efficacy and job engagement, thus, job challenges can lead to the development of self-efficacy and achieving successful outcomes. Additionally, self-efficacy can serve as a mediating factor between challenging job demands and job engagement. This implies that individuals who experience and overcome job challenges can develop higher self-efficacy, which, in turn, can influence their commitment and positive involvement in work (72).

Nurses with high levels of self-efficacy are more guided by their internal goals for their careers than nurses with low levels of self-efficacy (76). They can perform practical skills or tasks more effectively and better understand the reasoning behind their execution (77), which can help hospital administrators retain their nurses (78). When nurses continue to promote their job competence and demonstrate their professional commitment and achievement preferences in their career, their self-evaluation of their nursing careers improves (79). Assessing self-efficacy within the JD-R model will allow for a better understanding of job demands as challenges that can have positive effects on self-efficacy and job engagement.

**Figure 1:**
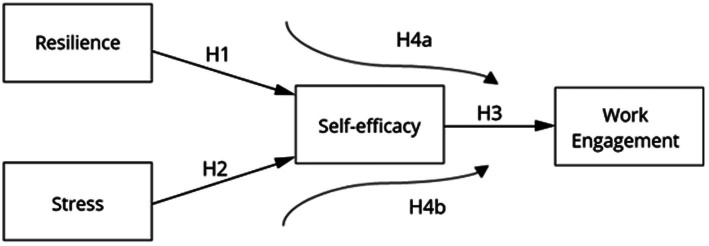
Theoretical model.

Based on the arguments presented, the following hypotheses are proposed (Figure 1):

*H1*: There is a positive relationship between resilience and self-efficacy.

*H2*: There is a negative relationship between stress and self-efficacy.

*H3*: There is a relationship between self-efficacy and work engagement.

*H4*a: Self-efficacy mediates the relationship between resilience and work engagement.

*H4*b: Self-efficacy mediates the relationship between stress and work engagement.

## 2. Methods

### 2.1. Design and participants

A cross-sectional explanatory study was designed, considering latent variables represented by a structural equation model (80). The sample size was evaluated using the effect size through the Soper electronic calculator (81), which takes into account the number of observed and latent variables in the structural equation model (SEM), the anticipated effect size (λ = 0.2), the desired statistical significance (α = 0.05), and the level of statistical power (1 – β = 0.90), which indicated a minimum sample size of 434 participants. The population consisted of 700 nurses, and the sample was selected through non-probabilistic sampling. The participants consisted of 459 Peruvian nurses. The mean age was *M* = 40.12 years (SD = 10.9), ranging from 22 to 68 years. Table 1 shows that the majority of nurses were female (62.3%), with a single marital status (34.2%), university education (81.7%), and temporary employment contracts (53.6%).

**Table 1:** Sociodemographic information.

Characteristic		*n*	%
Sex	Female	286	62.3
	Male	173	37.7
Marital Status	Married	112	24.4
	Living together	138	30.1
	Divorced	38	8.3
	Single	157	34.2
	Widowed	14	3.1
Academic formation	Specialty	44	9.6
	Postgraduate	34	7.4
	Technical	6	1.3
	University degree	375	81.7
Employment status	Indefinite work contract	46	10.0
	Temporary work contract	246	53.6
	Permanent appointment	108	23.5
	Replacement work contract	12	2.6
	Contract with service providers (third parties)	47	10.2

### 2.2. Procedure

The project was approved by the Ethics Committee of a Peruvian University (2022-CEUPeU-026). Afterwards, participants were invited to answer a questionnaire that was available online through Google Forms from September 2nd to December 30th, 2022. Before collecting data, confidentiality norms and protocols established in the Declaration of Helsinki were respected. Participants were informed about the research objective and gave their informed consent before starting.

#### 2.2.1. Instruments

##### 2.2.1.1. Work engagement

The Spanish version of the Brief Commitment Scale (UWES-9) was used to evaluate the work engagement of health professionals (82). This scale consists of nine items that are rated on a six-point Likert-type scale, ranging from “never” (0) to “always” (5). The scale focuses on three dimensions: vigor, dedication, and absorption. The internal consistency of the scale was determined using Cronbach’s alpha, and a variation from 0.84 to 0.92 was observed for the dimensions. The UWES-9 is a tool that has been adapted to Peruvian Spanish, showing adequate psychometric properties, and the internal consistency reliability measured by McDonald’s Omega was appropriate (ω = 0.85).

##### 2.2.1.2. Self-efficacy

The Spanish version of the General Self-efficacy Questionnaire (GSQ) (83) was used to measure self-efficacy, which is a simplified version of the General Self-efficacy Model by Schwarzer (84). This scale consists of 10 questions, with a minimum score of 10 points and a maximum of 40 points, which are rated on a Likert-type scale. Responses range from “Incorrect” (1 point) to “True” (4 points), depending on the perception of one’s own ability at that moment. The internal consistency of the scale was evaluated using Cronbach’s alpha coefficient, which obtained a value of 0.84, indicating good internal consistency. The GSQ has been adapted to Peruvian Spanish, reporting adequate psychometric properties, and its internal consistency measured by the categorical Omega coefficient was appropriate (ωcategorical = 0.79) (85).

##### 2.2.1.3. Resilience

The Brief Resilient Coping Scale (BRCS) adapted to Spanish in its unidimensional version was used to measure Spanish resilience (86). This scale focuses on the ability to manage stressful factors adaptively and consists of four items that are rated on a Likert-type scale ranging from 1 (not at all describing me) to 5 (describing me very well). Internal consistency was evaluated using the Composite Reliability Index and obtained a result of 0.70. The Peruvian Spanish adapted version was used, and the internal consistency reliability of the scale was estimated at 0.87 (87).

##### 2.2.1.4. Stress

The Perceived Stress Scale (PSS-4) in its Spanish version (88), adapted from the English version (89), was used to evaluate stress. The scale consists of four items, two of which are written positively (1 and 4) and two negatively (2 and 3). The scale is a Likert-type scale ranging from 0 (never) to 4 (very often). Reliability was evaluated using Cronbach’s alpha, which was adequate with a value of 0.74, and the omega coefficient, with a value of 0.78.

### 2.3. Statistical analysis

An initial analysis of possible common method bias was conducted, which arises due to the use of self-administered questionnaires in data collection. This bias refers to measurement error that can be introduced in the study due to the design of the data collection instrument (90, 91). To mitigate this effect, the statistical strategy of assessing common method variance (CMV) was employed through the Harman’s single-factor test. This method is based on the expectation that if there is common method bias, a single factor will emerge in the principal component analysis, explaining a majority proportion of the variance. In our case, we set the criterion that this factor should not account for more than 50% of the total variance (90).

The theoretical study model was analyzed using structural equation modeling with the MLR estimator, which is appropriate for numerical variables and is robust to deviations from inferential normality (92). Model fit was evaluated using the comparative fit index (CFI), root mean square error of approximation (RMSEA), and standardized root mean square residual (SRMR). CFI and TLI values greater than 0.90 (93), RMSEA values <0.08 (94), and SRMR values <0.08 (95) were used as the cutoffs for acceptable fit.

The software used was “R” version 4.1.2 and the “lavaan” library version 06–10 (96) was used.

## 3. Results

### 3.1. Common method variance

Table 2 displays that the variance accounted for 45%, indicating that the data set does not exhibit common method variance (CMV) through the Harman’s single-factor test, as the results indicated it was below the threshold (<50%) (90).

**Table 2:** Common method variance (CMV).

Component	Initial eigenvalues			Extraction sums of squared loadings		
	Total	% Var	Cumulative %	Total	% Var	Cumulative %
1	12.1	0.45	0.45	12.1	0.45	0.45
2	7.41	0.27	0.72			
3	1.96	0.07	0.80			
4	1.49	0.06	0.85			
5	0.78	0.03	0.88			
6	0.40	0.01	0.89			
7	0.37	0.01	0.91			
8	0.31	0.01	0.92			
9	0.25	0.01	0.93			
10	0.24	0.01	0.94			
11	0.21	0.01	0.94			
12	0.18	0.01	0.95			
13	0.16	0.01	0.96			
14	0.15	0.01	0.96			
15	0.13	0.00	0.97			
16	0.12	0.00	0.97			
17	0.11	0.00	0.98			
18	0.10	0.00	0.98			
19	0.09	0.00	0.98			
20	0.08	0.00	0.99			
21	0.08	0.00	0.99			
22	0.07	0.00	0.99			
23	0.06	0.00	0.99			
24	0.05	0.00	1.00			
25	0.04	0.00	1.00			
26	0.04	0.00	1.00			
27	0.03	0.00	1.00			

### 3.2. Preliminary analysis

Table 3 shows the descriptive results and the correlation matrix, which shows a high and significant positive correlation between job commitment and self-efficacy (*r* = 0.86, *p* < 0.01), as well as between self-efficacy and resilience (*r* = 0.74, *p* < 0.01). A high and significant positive correlation is also observed between resilience and job commitment (*r* = 0.72, *p* < 0.01). On the other hand, a moderate and significant negative correlation is observed between stress and job commitment (*r* = −0.32, *p* < 0.01), as well as between stress and self-efficacy (*r* = −0.30, *p* < 0.01), and between stress and resilience (*r* = −0.29, *p* < 0.01).

**Table 3:** Descriptive statistics, internal consistencies, and correlations for the study variables.

Variables	*M*	*SD*	1	2	3	4
1. Work engagement	32.05	9.88	1			
2. Self-efficacy	25.43	7.31	0.86**	1		
3. Resilience	13.50	3.20	0.72**	0.74**	1	
4. Stress	8.20	2.82	−0.32**	−0.30**	−0.29**	1

M, mean; SD, standard deviation. **Indicates *p* < 0.01.

### 3.3. Analysis of the theoretical model

**Figure 2:**
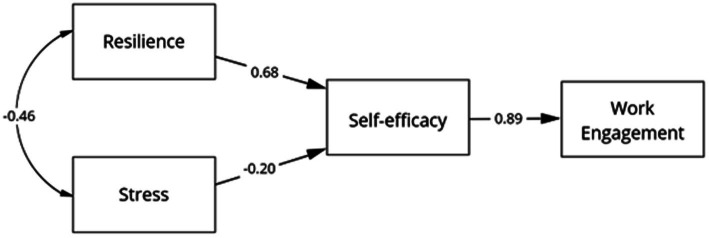
Result of the explanatory structural of work commitment.

In the analysis of the theoretical model, an adequate fit was obtained (Figure 2), *χ*^2^ = 994.29, *df* = 316, *p* < 0.001, CFI = 0.92, TLI = 0.91, RMSEA = 0.07 (CI: 0.06–0.07), SRMR = 0.07. With this result, H1 is confirmed regarding the influence of resilience (*β* = 0.68, *p* < 0.001) and stress (*β* = −0.20, *p* < 0.05) on job satisfaction. Also, the positive relationship between self-efficacy and job engagement is confirmed (*β* = 0.89, *p* < 0.001).

### 3.4. Mediation model

For the mediation analysis, bootstrapping with 5,000 iterations was used and the results are shown in Table 4. The mediating role of self-efficacy in the relationship between resilience and job commitment was confirmed, *β* = 0.91, *p* = <0.001 (*H4*a). Similarly, the mediating role between stress and job commitment was confirmed, *β* = −0.66, *p* = 0.04 (*H4*b).

**Table 4:** Research hypotheses on indirect effects and their estimates.

				95%CI	
Hypothesis	Path in the model	β	*p*	LL	UL
Hypothesis 4a	Resilience → Self-efficacy→ Job commitment	0.91	<0.001	0.73	1.05
Hypothesis 4b	Stress → Self-efficacy → Job commitment	−0.66	0.04	−1.53	−1.53

## 4. Discussion

Work-related self-efficacy is a key aspect of personal resources that is associated with self-directed motivation, positive expectations of success, and a greater capacity to withstand difficult work situations. Self-efficacy is a predictor of positive states such as work engagement, especially in demanding environments, as well as the ability to successfully cope with or recover from adverse circumstances. Additionally, self-efficacy can assist healthcare professionals in addressing challenges and performing their job more effectively. The Job Demands-Resources model emphasizes the role of personal resources in work engagement and performance, and their ability to reduce the negative impact of work demands such as stress. Thus, self-efficacy is an important resource for nurses that can help them feel more capable of facing challenges and improving their professional commitment and self-evaluation of their nursing career. Therefore, the aim of this research is to evaluate the mediating role of self-efficacy in the relationship between resilience, stress, and work engagement.

The present study has demonstrated the existence of a positive influence between resilience and self-efficacy in nurses, which aligns with the Job Demands-Resources (JD-R) model. According to this model, resilience has a significant positive direct effect on nurses’ self-efficacy, and in turn, greater self-efficacy contributes to a higher sense of resilience in the workplace (23, 97–99). Self-efficacy helps nurses cope with clinical challenges, which in turn can develop their resilience. This finding is important because resilience is a key factor for nurses to handle stressful situations and prevent emotional exhaustion, mental fatigue, lack of motivation, and intention to leave (100). Nurses with higher levels of resilience are more likely to use job resources to cope with job stressors and improve their emotional control (60–62). Therefore, it is crucial to understand resilience and provide support to develop programs that help nurses to be and remain resilient (101). Challenging work environments, psychological emptiness, decreased perception of well-being, and dissonance have been identified as factors that contribute to resilience in nurses (50). Different strategies, such as cognitive reframing, hardiness, grounding connections, work-life balance, and reconciliation, have been proposed to promote resilience in this professional group. Additionally, understanding the positive influence between resilience and self-efficacy can be useful in teaching/learning practices that promote nurse retention. Nurses with higher levels of self-efficacy in their early career years, who perceive they can perform well, are more likely to view difficult tasks as something to master rather than avoid (23). Therefore, understanding the relationship between resilience and self-efficacy can be valuable for fostering a positive work environment and retaining nursing professionals.

Furthermore, the negative influence of stress on nurses’ self-efficacy has been demonstrated, which is consistent with the JD-R model and the Demand-Resources theory of work. The results indicate that stress increases when the person has less control over the situation and lower self-efficacy (102–105). Although there are studies that show opposite results, where people with higher self-efficacy experience more stress, this result could be explained through the determining role of personality in the relationship between self-efficacy and stress, as several studies have pointed out (106, 107). Therefore, it is recommended to continue researching the role of personality and mood variables in the relationship between self-efficacy and stress. On the other hand, self-efficacy has been identified as a protective factor for nurses experiencing stress during health crises, which reinforces the importance of supporting self-efficacy as a work resource to improve their mental health and well-being (102, 105). Workload, the nature of nursing work, family, expectations, interpersonal relationships, and patient contact are the main sources of stress for nurses (108). Self-efficacy is formed through individual experience, and as a person works more and overcomes severe challenges with persistence and hard work (109). Work stress also affects nurses’ resilience, as perceived high levels of stress decrease resilience (98). Therefore, it is important for nurses to learn how to manage their stress and focus on personal and environmental stressors to improve their resilience.

The study also demonstrated a positive influence between self-efficacy and work engagement among nurses. These findings are consistent with the JD-R model and agree with other studies that have found a significant association between self-efficacy and work engagement (31, 110). This is because nurses with high levels of self-efficacy are able to effectively manage their work environment, cope with challenges, and mobilize additional resources if necessary. This results in greater effort, motivation, and persistence at work, which in turn leads to greater dedication, absorption, and vigor, i.e., higher work engagement (13, 24, 32, 111). Thus, when a nurse feels engaged in their work, they experience greater energy and are absorbed in their work. In addition, they feel proud of their work and consider their work to have meaning and are involved in their position. This leads to greater personal and professional satisfaction in their work, resulting in a stronger affective bond with the institution and a lower intention to leave work (13, 112, 113). The findings suggest that self-efficacy is a valuable work resource to support work engagement among nurses and improve their satisfaction and well-being at work.

Another finding indicated that self-efficacy is an important factor that mediates the relationship between resilience and work engagement in nurses. According to the JD-R model, resilience and self-efficacy are considered as job resources that positively influence work engagement and psychological well-being of nurses (114). Previous studies have found that resilience improves nurses’ work engagement and workload can positively predict burnout (60–62, 115). Therefore, it is important to further investigate the mediation of self-efficacy in the relationship between resilience and work engagement to better understand how to improve mental health and well-being of nurses in the work environment. Additionally, the JD-R model highlights the importance of understanding job resources that facilitate resilience and positive adaptation at work to improve the mental health of individuals (58).Therefore, it is necessary to explore the moderating roles of self-efficacy and other factors in the relationship between work engagement and resilience to better understand how to improve mental health and well-being of nurses in the work environment.

Furthermore, the results of this study have confirmed that self-efficacy plays an important role in mediating the relationship between stress and job engagement in nurses. According to the JD-R model, stress is considered a job demand, which can have negative effects on workers’ health and well-being (39). However, nurses with high self-efficacy can handle stress more effectively by adopting positive, problem-focused coping strategies (116). As a result, they are able to maintain good job engagement, with higher motivation, dedication, and absorption in their work (24, 32). On the other hand, nurses with low self-efficacy may experience doubts and negative emotions in situations of job stress, which can decrease their job efficiency and reduce their job engagement (117). Therefore, it is important for nurses to strengthen their self-efficacy to improve their ability to handle stress and maintain positive job engagement in their work.

### 4.1. Implications

Nursing managers and administrators should consider the importance of resilience, stress, job engagement, and self-efficacy in registered nurses during their early career and develop effective strategies to improve them. To increase resilience and self-efficacy, it is necessary to encourage the acquisition or improvement of psychological resources and provide tangible, emotional, informational, or companion support to nurses to reduce stress. Self-efficacy and job engagement are important factors in nurses’ affective organizational commitment. Success experiences, overcoming obstacles, verbal persuasion, and good mood are important sources of information on personal effectiveness. Additionally, higher job resources allow for high levels of dedication, vigor, and absorption in nursing staff. Social support and coworker support also play an important role in nurses’ resilience, so it is important to improve communication and the work environment to encourage a supportive and collaborative environment.

Hospital administrators can strengthen family and social support for nurses by establishing, for example, a psychological counseling department. It is also important to note that proper attention to resilience, self-efficacy, job engagement, and stress reduction in nurses also has significant implications for patient care. A nurse with adequate resilience and self-efficacy and high job engagement is more likely to provide quality care and have a lower error rate in their work. Furthermore, a healthy and positive work environment can also improve nurses’ motivation and job satisfaction, which in turn can have a positive impact on their performance and the quality of care they provide to patients.

Therefore, it is important for nursing managers and administrators to invest in training and skills development to improve nurses’ resilience, self-efficacy, and job engagement. This may include implementing mentoring programs, providing emotional support, and creating opportunities for outdoor activities. Additionally, it is important to establish well-being mechanisms, such as an adequate leave system, to reduce emotional fatigue and stress in nurses.

### 4.2. Limitations

Despite the valuable findings obtained in this study, there are limitations that should be considered. First, the sample used may not be representative of all nurses in Peru, so it is important to expand the research to a larger sample to confirm the results. In addition, although this study used a quantitative survey, it would be useful to combine quantitative data with qualitative data in future research to obtain a deeper and more reliable understanding of the factors influencing nurses’ engagement. The cross-sectional design used in this study does not allow exploration of the trend of variables over time, so it would be justified to use a longitudinal design in future research to investigate the change in nurses. Furthermore, although the study used self-report questionnaires, these questionnaires may be biased, and it would be useful to use more objective measurement tools in future research.

## 5. Conclusion

In conclusion, self-efficacy is a key factor in the relationship between resilience and stress on nurses’ job engagement. High levels of self-efficacy help nurses feel more prepared to cope with job demands and improve their engagement and performance at work. Research has shown that self-efficacy is an important personal resource for the health and well-being of workers and can help prevent burnout and other work-related health problems.

## Data availability statement

The raw data supporting the conclusions of this article will be made available by the authors, without undue reservation.

## Ethics statement

The studies involving human participants were reviewed and approved by Ethics Committee of the Peruvian Union University (2022-CEUPeU-026). The patients/participants provided their written informed consent to participate in this study.

## Author contributions

EC-A, WM-G, and MZ-F participated in the conceptualization of the idea. WM-G, MM-G, and AR-C were in charge of the methodology and software. LS-S, AR-C, and WM-G done the validation, formal analysis, and research. WM-G, EC-A, and MZ-F commissioned the data curation and resources. LS-S, WM-G, EC-A, and MM-G carried out the writing of the first draft, review and editing, visualization and supervision. All authors contributed to the article and approved the submitted version.

## Conflict of interest

The authors declare that the research was conducted in the absence of any commercial or financial relationships that could be construed as a potential conflict of interest.

## Publisher’s note

All claims expressed in this article are solely those of the authors and do not necessarily represent those of their affiliated organizations, or those of the publisher, the editors and the reviewers. Any product that may be evaluated in this article, or claim that may be made by its manufacturer, is not guaranteed or endorsed by the publisher.
